# Supplementary material for: CHEcking Diagnostic Differential Ability of Real Baseline Variables and Frailty Scores in Tolerance of Anti-Cancer Systemic Therapy in OldEr Patients (CHEDDAR-TOASTIE)
Source: Cancers (Basel). 2025 Oct 13;17(20):3303. doi: 10.3390/cancers17203303 (PMC12564391; doi:10.3390/cancers17203303)
Supplement: Supplementary file 1 [file cancers-17-03303-s001.zip › cancers-3859831-supplementary.pdf]

Supplementary Files

List S1. List of variables available:

1) Demographics

Site, Patient number, Patient Study ID, Date of clinic visit, Age at time of clinic visit, Sex, Height (m), Weight (kg), BMI, WHO/ECOG PS (0-4), Karnofsky PS (%), Cancer diagnosis, Cancer type? 1 - upper gastrointestinal 2- gynae 3-lung 4-breast 5- lower gastrointestinal 6-HPB 7-urology 8-other, Cancer Stage? (I - IV), TNM staging, Site of mets (please list), ExcludeNonchemo, regimenrefined, Chemotherapy regime (please list all drugs), No. of chemotherapy drugs, Intention of chemotherapy 1-neo-adjuvant 2-adjuvant 3-palliative , Date of first clinic visit (dd/mm/yyyy), Dose prescribed for cycle one: 1- full 2-reduced, If reduced, to what %?, Reason for dose reduction?, Hb, Hb normal range, Platelets, Platelets normal range, Creatinine, Creatinine normal range, Cr Cl, CrCl type, Tumour markers Which?, Elevated? y/n, Bilirubin, Bili normal range, Albumin, Albumin normal range, Neutrophils, Neutrophils normal range, Lymphocytes, Lymphocytes normal range, y/n, Total number of co-mobidities, MI (History of definite or probable MI (EKG changes and/or enzyme changes) 0-no 1-yes , CHF (Exertional or paroxysmal nocturnal dyspnea and has responded to digitalis, diuretics, or afterload reducing agents) 0-no 1-yes, PVD (Intermittent claudication or past bypass for chronic arterial insufficiency, history of gangrene or acute arterial insufficiency, or untreated thoracic or abdominal aneurysm (≥6 cm)) 0-no 1-yes, CVA or TIA 0-no 1-yes, Dementia (Chronic cognitive deficit) 0-no 1-yes, COPD 0-no 1-yes, CTD (connective tissue disease)0-no 1-yes, Peptic Ulcer Disease (Any history of treatment for ulcer disease or history of ulcer bleeding) 0-no 1-yes, Liver disease (Severe = cirrhosis and portal hypertension with variceal bleeding history, moderate = cirrhosis and portal hypertension but no variceal bleeding history, mild = chronic hepatitis (or cirrhosis without portal hypertension)) 0-none 1-mild 3-moderate to severe, Diabetes 0-none or diet controlled 1-unomplicated 2-end organ damage, Hemiplegia 0-no 1-yes, AIDS 0-no 1-yes, Lymphoma 0-no 1-yes, Leukaemia 0-no 1-yes, Number of regular medications?, Any formal/qualatitive frailty assessment completed? y/n, If yes.... Please give details of assessment type? , ,

2) AHQ:

- hearing? 0. normal 1. impaired?,
- Any falls in the last 6 months 0. none 1. 1 or more,
- in the past 4 weeks has patients physical or emotional health interfered with social activites eg visiting friends? 1. all the time 2. most of the time 3. some of the time 4. a little of the time 5. none of the time,

- Patients ability to take own medications? 1. without help 2. with some help (pre-prepared/reminders) 3. unable,
- Does patient's health limit them walking one block? 1. limited a lot 2. limited a little 3. no limitations,
- How many regular medications does the patient take?,
- Any weight loss past 3 months 1. >3kg 2. 1-3kg 3. don't know 4. no weight loss,
- Describe mobility 1. bed/chair bound 2.can get out of chair/bet but doesn't get out 3. gets out,
- Has food intake declined in the past 3 months? 1. severe decrease 2. moderate decrease 3. no decrease,
- Any psychological issues? 1. severe dementia/depression 2. mild dementia 3. no problems,
- Rockwood CFS (see attached guide) 1-9,
- Time taken to ask these questions (mins)

3) Patient Questionnaire:

Site, Patient number, Patient ID, Q1 Marital Status 1 - single 2 - married 3 - widow/widower, Q2 Living situation 1-alone 2-with partner/family 3-I am carer for partner/family, Q3. When you need help, for example someone to help you with your shopping or take you to a hospital appointment, do you have someone you can you count on to help you with this 1-always 2- sometimes 3-never 4-I never need help, Q4 Do you smoke? 1-yes 2-no used to, 3-no never, Q5 How many hospital admissions have you had in the past 12 months? 1- 0 2-1-2 3-3 or more, Q6 In comparison with people of the same age how do you consider your health? 1. not as good 2. as good. 3 better 4. don't know , Q7. How likely do you think it is that a side effect from chemotherapy will cause you to stay in hospital for one night or longer or stop treatment? 1. unlikely 2. no very likely 3. quite likely 4. very likely, riskavailable, Q7 estimated risk % (as percentage), Q8 We would like to know how good or bad your health is TODAY (0-100), Q9 How long did it take you to fill in this questionnaire 1-<5mins 2-5-10mins 3->10mins

4) Researcher's Risk

- Q1 (estimated risk of significant toxicity) 1. low 2. low-med 3. med-high 4. high,
- Q2 (estimated risk %) as a percentage

List S2. List of non-base R packages used

readxl

tidyverse

finalfit

Patchwork

mice

caret

pROC

Cutpointr

glmnet

ranger

Table S1. Data partition for internal validation. Training, validation and testing cohorts.

G3+ toxicities:	**TRUE**	**FALSE**	**Total (% with true)**
Training	47	175	222 (21.2%)
Validation	11	37	48 (22.9%)
Testing	12	38	50 (24.0%)

Table S2. Baseline characteristics of all patients prior to removing patients with missing outcomes. (*Green highlights significant differences between presence and absence of severe chemotherapy related toxicities)*

**Dependent: Severe chemotherapy related toxicities**		**No**	**Yes**	**(Missing)**	**Total**	**p**
Total N (%)		251 (72.3)	71 (20.5)	25 (7.2)	347	
age	Mean (SD)	72.6 (4.8)	73.2 (5.0)	75.8 (6.7)	73.0 (5.0)	0.356
Sex	Female	124 (49.4)	32 (45.1)	11 (44.0)	167 (48.1)	0.66
	Male	126 (50.2)	38 (53.5)	14 (56.0)	178 (51.3)	
	(Missing)	1 (0.4)	1 (1.4)	0 (0.0)	2 (0.6)	
BMI	Mean (SD)	27.2 (5.1)	26.6 (5.3)	25.4 (3.8)	26.9 (5.0)	0.423
WHO Performance Status (0-4)	Mean (SD)	0.7 (0.7)	1.0 (0.7)	1.1 (0.6)	0.8 (0.7)	0.004
Cancer Type	Upper GI	63 (25.1)	17 (23.9)	8 (32.0)	88 (25.4)	0.561
	Gynaecological	37 (14.7)	8 (11.3)	3 (12.0)	48 (13.8)	
	Lung	16 (6.4)	5 (7.0)	5 (20.0)	26 (7.5)	
	Breast	21 (8.4)	6 (8.5)	2 (8.0)	29 (8.4)	
	Lower GI	60 (23.9)	13 (18.3)	3 (12.0)	76 (21.9)	
	HPB	14 (5.6)	9 (12.7)	2 (8.0)	25 (7.2)	
	Urological	27 (10.8)	7 (9.9)	0 (0.0)	34 (9.8)	
	Other	12 (4.8)	5 (7.0)	1 (4.0)	18 (5.2)	
	(Missing)	1 (0.4)	1 (1.4)	1 (4.0)	3 (0.9)	
Cancer Stage	1	10 (4.0)	2 (2.8)	2 (8.0)	14 (4.0)	0.792
	2	38 (15.1)	9 (12.7)	2 (8.0)	49 (14.1)	
	3	75 (29.9)	25 (35.2)	3 (12.0)	103 (29.7)	
	4	88 (35.1)	29 (40.8)	10 (40.0)	127 (36.6)	
	(Missing)	40 (15.9)	6 (8.5)	8 (32.0)	54 (15.6)	
Presence of metastases	FALSE	62 (35.2)	20 (35.7)	1 (5.0)	83 (32.9)	1
	TRUE	114 (64.8)	36 (64.3)	19 (95.0)	169 (67.1)	
Number of chemotherapy drugs	Mean (SD)	2.1 (0.6)	2.2 (0.5)	1.9 (0.4)	2.1 (0.6)	0.183
Intention of chemotherapy	Neo-adjuvant	73 (29.1)	21 (29.6)	2 (8.0)	96 (27.7)	0.874

	Adjuvant	61 (24.3)	15 (21.1)	4 (16.0)	80 (23.1)	
	Palliative	116 (46.2)	34 (47.9)	18 (72.0)	168 (48.4)	
	(Missing)	1 (0.4)	1 (1.4)	1 (4.0)	3 (0.9)	
Was dose reduced?	FALSE	161 (64.9)	39 (55.7)	16 (66.7)	216 (63.2)	0.205
	TRUE	87 (35.1)	31 (44.3)	8 (33.3)	126 (36.8)	
Low baseline haemoglobin	FALSE	158 (65.8)	33 (51.6)	10 (47.6)	201 (61.8)	0.051
	TRUE	82 (34.2)	31 (48.4)	11 (52.4)	124 (38.2)	
High baseline haemoglobin	FALSE	238 (99.2)	64 (100.0)	21 (100.0)	323 (99.4)	1
	TRUE	2 (0.8)			2 (0.6)	
Low platelet count at baseline	FALSE	240 (96.4)	68 (97.1)	23 (95.8)	331 (96.5)	1
	TRUE	9 (3.6)	2 (2.9)	1 (4.2)	12 (3.5)	
High platelet count at baseline	FALSE	208 (83.5)	58 (82.9)	18 (72.0)	284 (82.6)	1
	TRUE	41 (16.5)	12 (17.1)	7 (28.0)	60 (17.4)	
Low creatinine at baseline	FALSE	205 (82.3)	53 (75.7)	22 (91.7)	280 (81.6)	0.284
	TRUE	44 (17.7)	17 (24.3)	2 (8.3)	63 (18.4)	
High creatinine at baseline	FALSE	226 (90.8)	58 (82.9)	21 (87.5)	305 (88.9)	0.098
	TRUE	23 (9.2)	12 (17.1)	3 (12.5)	38 (11.1)	
Low creatinine clearance at baseline	FALSE	24 (11.6)	6 (9.2)	1 (4.2)	31 (10.5)	0.761
	TRUE	183 (88.4)	59 (90.8)	23 (95.8)	265 (89.5)	
High creatinine clearance at baseline	FALSE	203 (98.1)	64 (98.5)	24 (100.0)	291 (98.3)	1
	TRUE	4 (1.9)	1 (1.5)		5 (1.7)	
High bilirubin count at baseline	FALSE	236 (95.2)	69 (100.0)	23 (95.8)	328 (96.2)	0.132
	TRUE	12 (4.8)		1 (4.2)	13 (3.8)	
Low albumin count at baseline	FALSE	184 (74.2)	51 (72.9)	19 (79.2)	254 (74.3)	0.944
	TRUE	64 (25.8)	19 (27.1)	5 (20.8)	88 (25.7)	
Low neutrophils count at baseline	FALSE	246 (99.2)	70 (100.0)	24 (100.0)	340 (99.4)	1
	TRUE	2 (0.8)			2 (0.6)	
High neutrophils count at baseline	FALSE	208 (83.9)	50 (71.4)	19 (79.2)	277 (81.0)	0.03
	TRUE	40 (16.1)	20 (28.6)	5 (20.8)	65 (19.0)	

Low lymphocytes count at baseline	FALSE	203 (81.9)	56 (80.0)	20 (83.3)	279 (81.6)	0.858
	TRUE	45 (18.1)	14 (20.0)	4 (16.7)	63 (18.4)	
High lymphocytes count at baseline	FALSE	245 (98.8)	70 (100.0)	24 (100.0)	339 (99.1)	0.822
	TRUE	3 (1.2)			3 (0.9)	
Presence of comorbidities	FALSE	86 (34.7)	18 (25.7)	9 (37.5)	113 (33.0)	0.205
	TRUE	162 (65.3)	52 (74.3)	15 (62.5)	229 (67.0)	
History of definite or probable myocardial infarction	FALSE	217 (87.9)	64 (91.4)	21 (87.5)	302 (88.6)	0.536
	TRUE	30 (12.1)	6 (8.6)	3 (12.5)	39 (11.4)	
Congestive Heart Failure	FALSE	243 (98.4)	69 (98.6)	24 (100.0)	336 (98.5)	1
	TRUE	4 (1.6)	1 (1.4)		5 (1.5)	
Peripheral Vascular Disease	FALSE	241 (97.6)	68 (97.1)	23 (95.8)	332 (97.4)	1
	TRUE	6 (2.4)	2 (2.9)	1 (4.2)	9 (2.6)	
History of cardiovascular accident	FALSE	235 (95.1)	63 (90.0)	24 (100.0)	322 (94.4)	0.189
	TRUE	12 (4.9)	7 (10.0)		19 (5.6)	
History of dementia	FALSE	245 (99.2)	70 (100.0)	24 (100.0)	339 (99.4)	1
	TRUE	2 (0.8)			2 (0.6)	
History of COPD	FALSE	232 (93.9)	67 (95.7)	20 (83.3)	319 (93.5)	0.781
	TRUE	15 (6.1)	3 (4.3)	4 (16.7)	22 (6.5)	
History of Connect Tissue Disease	FALSE	242 (98.0)	68 (97.1)	24 (100.0)	334 (97.9)	1
	TRUE	5 (2.0)	2 (2.9)		7 (2.1)	
History of Peptic Ulcer Disease or bleeding	FALSE	244 (98.8)	69 (98.6)	24 (100.0)	337 (98.8)	1
	TRUE	3 (1.2)	1 (1.4)		4 (1.2)	
History of Liver disease	FALSE	245 (99.2)	69 (98.6)	24 (100.0)	338 (99.1)	1
	TRUE	2 (0.8)	1 (1.4)		3 (0.9)	
History of diabetes	FALSE	220 (89.1)	59 (84.3)	21 (87.5)	300 (88.0)	0.379
	TRUE	27 (10.9)	11 (15.7)	3 (12.5)	41 (12.0)	

History of hemiplegia	FALSE	241 (97.6)	68 (97.1)	24 (100.0)	333 (97.7)	1
	TRUE	6 (2.4)	2 (2.9)		8 (2.3)	
History of AIDS	FALSE	246 (99.6)	70 (100.0)	24 (100.0)	340 (99.7)	1
	TRUE	1 (0.4)			1 (0.3)	
Number of regular medications taken	Mean (SD)	3.7 (2.9)	4.4 (3.0)	4.1 (3.5)	3.9 (3.0)	0.106
Researcher's estimated risk of significant toxicity	Low	33 (13.1)	9 (12.7)	1 (4.0)	43 (12.4)	0.183
	Low-medium	118 (47.0)	26 (36.6)	14 (56.0)	158 (45.5)	
	Medium-high	65 (25.9)	28 (39.4)	5 (20.0)	98 (28.2)	
	High	5 (2.0)	2 (2.8)	1 (4.0)	8 (2.3)	
	(Missing)	30 (12.0)	6 (8.5)	4 (16.0)	40 (11.5)	
Researcher's estimated risk of significant toxicity In percentage	Mean (SD)	34.1 (19.7)	38.7 (20.1)	36.6 (19.2)	35.2 (19.8)	0.099
AHQ: presence of impaired hearing	FALSE	194 (77.6)	51 (72.9)	14 (58.3)	259 (75.3)	0.504
	TRUE	56 (22.4)	19 (27.1)	10 (41.7)	85 (24.7)	
AHQ: Fall(s) in last 6 months	FALSE	210 (84.0)	60 (85.7)	18 (75.0)	288 (83.7)	0.871
	TRUE	40 (16.0)	10 (14.3)	6 (25.0)	56 (16.3)	
AHQ: Interference of social activities due to health	All the time	10 (4.0)	10 (14.1)	0 (0.0)	20 (5.8)	0.004
	Most of the time	28 (11.2)	9 (12.7)	4 (16.0)	41 (11.8)	
	Some of the time	38 (15.1)	15 (21.1)	2 (8.0)	55 (15.9)	
	A little of the time	21 (8.4)	8 (11.3)	3 (12.0)	32 (9.2)	
	none of the time	153 (61.0)	28 (39.4)	15 (60.0)	196 (56.5)	
	(Missing)	1 (0.4)	1 (1.4)	1 (4.0)	3 (0.9)	
AHQ: Ability to take own medications	Without help	240 (95.6)	62 (87.3)	21 (84.0)	323 (93.1)	0.035
	With some help or reminders	9 (3.6)	6 (8.5)	3 (12.0)	18 (5.2)	
	Unable	1 (0.4)	2 (2.8)	0 (0.0)	3 (0.9)	
	(Missing)	1 (0.4)	1 (1.4)	1 (4.0)	3 (0.9)	

AHQ: Effect of health in walking one block	Limited a lot	10 (4.0)	6 (8.5)	0 (0.0)	16 (4.6)	0.001
	Limited a little	21 (8.4)	16 (22.5)	11 (44.0)	48 (13.8)	
	No limitations	219 (87.3)	48 (67.6)	13 (52.0)	280 (80.7)	
	(Missing)	1 (0.4)	1 (1.4)	1 (4.0)	3 (0.9)	
AHQ: Weight loss in the past 3 months	Yes	130 (51.8)	48 (67.6)	12 (48.0)	190 (54.8)	0.004
	No	111 (44.2)	16 (22.5)	8 (32.0)	135 (38.9)	
	(Missing)	10 (4.0)	7 (9.9)	5 (20.0)	22 (6.3)	
AHQ: Describe mobility	Bed or chair bound	1 (0.4)	0 (0.0)	0 (0.0)	1 (0.3)	<0.001
	Can get out but doesn't	7 (2.8)	11 (15.5)	2 (8.0)	20 (5.8)	
	Gets out	242 (96.4)	59 (83.1)	22 (88.0)	323 (93.1)	
	(Missing)	1 (0.4)	1 (1.4)	1 (4.0)	3 (0.9)	
AHQ: Decline in food intake in the past 3 months	Severe decrease	36 (14.3)	15 (21.1)	2 (8.0)	53 (15.3)	0.004
	Moderate decrease	76 (30.3)	32 (45.1)	10 (40.0)	118 (34.0)	
	No decrease	138 (55.0)	23 (32.4)	12 (48.0)	173 (49.9)	
	(Missing)	1 (0.4)	1 (1.4)	1 (4.0)	3 (0.9)	
Rockwood_CFS	Mean (SD)	2.5 (1.2)	3.0 (1.3)	3.3 (1.5)	2.6 (1.3)	0.004
Patient Questionnaire: Marital Status	Single	42 (16.7)	11 (15.5)	2 (8.0)	55 (15.9)	0.198
	Married	162 (64.5)	49 (69.0)	16 (64.0)	227 (65.4)	
	Widowed	27 (10.8)	9 (12.7)	3 (12.0)	39 (11.2)	
	Other	15 (6.0)	0 (0.0)	0 (0.0)	15 (4.3)	
	(Missing)	5 (2.0)	2 (2.8)	4 (16.0)	11 (3.2)	
Patient Questionnaire: Living situation	Alone	67 (26.7)	16 (22.5)	4 (16.0)	87 (25.1)	0.811
	With partner/family	170 (67.7)	51 (71.8)	16 (64.0)	237 (68.3)	
	Carer for partner/family	8 (3.2)	3 (4.2)	1 (4.0)	12 (3.5)	
	Other	1 (0.4)	0 (0.0)	0 (0.0)	1 (0.3)	
	(Missing)	5 (2.0)	1 (1.4)	4 (16.0)	10 (2.9)	

Patient Questionnaire: Is there someone to help you with shopping or appointments?	Always	171 (68.1)	52 (73.2)	15 (60.0)	238 (68.6)	0.258
	Sometimes	23 (9.2)	9 (12.7)	4 (16.0)	36 (10.4)	
	Never	24 (9.6)	2 (2.8)	2 (8.0)	28 (8.1)	
	I do not need help	27 (10.8)	7 (9.9)	0 (0.0)	34 (9.8)	
	(Missing)	6 (2.4)	1 (1.4)	4 (16.0)	11 (3.2)	
Patient Questionnaire: Smoking status	Current	24 (9.6)	8 (11.3)	2 (8.0)	34 (9.8)	0.797
	Ex-smoker	116 (46.2)	30 (42.3)	15 (60.0)	161 (46.4)	
	Never	106 (42.2)	32 (45.1)	4 (16.0)	142 (40.9)	
	(Missing)	5 (2.0)	1 (1.4)	4 (16.0)	10 (2.9)	
Patient Questionnaire: comparison of health to others of similar age	Not as good	24 (9.6)	14 (19.7)	5 (20.0)	43 (12.4)	0.007
	As good	102 (40.6)	19 (26.8)	8 (32.0)	129 (37.2)	
	Better	111 (44.2)	31 (43.7)	6 (24.0)	148 (42.7)	
	Don't know	7 (2.8)	6 (8.5)	2 (8.0)	15 (4.3)	
	(Missing)	7 (2.8)	1 (1.4)	4 (16.0)	12 (3.5)	
Patient Questionnaire: Consideration of likelihood of side effect from chemotherapy will lead to hospital stay	Unlikely	116 (46.2)	24 (33.8)	8 (32.0)	148 (42.7)	0.209
	Not very likely	110 (43.8)	36 (50.7)	10 (40.0)	156 (45.0)	
	Quite likely	16 (6.4)	8 (11.3)	2 (8.0)	26 (7.5)	
	Very likely	2 (0.8)	1 (1.4)	1 (4.0)	4 (1.2)	
	(Missing)	7 (2.8)	2 (2.8)	4 (16.0)	13 (3.7)	
Patient Questionnaire: Consideration of	Mean (SD)	23.5 (22.7)	29.6 (21.6)	23.2 (25.8)	24.8 (22.7)	0.05

likelihood of side effect from chemotherapy will lead to hospital stay in percentage						
Patient Questionnaire: How does the patient rate their health today in percentage	Mean (SD)	66.5 (26.0)	62.6 (24.6)	68.6 (23.1)	65.8 (25.6)	0.257

Table S3. Model performance metrics from training, validation and testing datasets.

TRAIN

	Logistic Regression								LASSO								Random Forest							
	Selected clinical variables				Significant Variables				Selected clinical variables				Significant Variables				Selected clinical variables				Significant Variables			
	CCA	Imp	Up-sample	Imp with Up-sample	CCA	Imp	Up-sample	Imp with Up-sample	CCA	Imp	Up-sample	Imp with Up-sample	CCA	Imp	Up-sample	Imp with Up-sample	CCA	Imp	Up-sample	Imp with Up-sample	CCA	Imp	Up-sample	Imp with Up-sample
Accuracy	0.7740	0.7928	0.6647	0.6782	0.8046	0.7883	0.6712	0.6552	0.5941	0.5675	0.6655	0.6314	0.5553	0.6216	0.6268	0.6143	0.9494		1		0.8626		0.9706	
95% CI	0.7111-0.829	0.7335-0.8441	0.6113-0.7151	0.6263-0.7270	0.7378-0.8607	0.7286-0.8401	0.6144-0.7245	0.6026-0.7050	0.5163-0.6686	0.4996-0.6337	0.6073-0.7201	0.5785-0.6821	0.4749-0.6291	0.5543-0.6857	0.5677-0.6832	0.5611-0.6656	0.9062 - 0.9766		0.9865-1.000		0.8039-0.9091		0.9429-0.9872	
p-value	0.6045	0.4735	<0.0001	<0.0001	0.1608	0.5390	<0.0001	<0.0001	1	1	<0.0001	<0.0001	1	1	0.002	<0.0001	1.94E-11		1		0.0006		<2e-16	
Balanced accuracy	0.5358	0.5418	0.6646	0.6782	0.6107	0.5467	0.6731	0.6552	0.6646	0.6168	0.6594	0.6314	0.6109	0.6433	0.6218	0.6143	0.8929		1		0.7093		0.9706	
Sensitivity	0.1087	0.1064	0.6706	0.6954	0.25	0.1277	0.6688	0.6437	0.5344	0.5314	0.5802	0.6457	0.5038	0.6057	0.5573	0.6400	0.7857		1		0.4186		0.9853	
Specificity	0.9630	0.9771	0.6585	0.6609	0.9702	0.9657	0.6738	0.6667	0.7949	0.7021	0.7386	0.6171	0.7179	0.6809	0.6863	0.5886	1		1		1		0.9559	
PPV	0.4546	0.5556	0.6706	0.6722	0.7143	0.5000	0.6913	0.6588	0.8974	0.8692	0.6552	0.6278	0.8571	0.8760	0.6033	0.6087	1		1		1		0.9571	
NPV	0.2212	0.8028	0.6585	0.6845	0.8125	0.8048	0.6507	0.6517	0.3370	0.2870	0.6726	0.6353	0.3011	0.3168	0.6442	0.6205	0.9379		1		0.8476		0.9848	
AUC	0.7116	0.7046	0.7003	0.7031	0.7608	0.7293	0.7833	0.7429	0.6646	0.6168	0.6594	0.6314	0.6109	0.6433	0.6218	0.6143	0.8929		1		0.7093		0.9706	
AUC 95%CI	0.6273-0.796	0.6197-0.7895	0.6441-0.7565	0.6481-0.7581	0.6708-0.8509	0.6450-0.7293	0.7314-0.8351	0.6921-0.7938	0.5874-0.7418	0.5410-0.6925	0.6044-0.7143	0.5808-0.6821	0.5274-0.6943	0.5668-0.7198	0.5653-0.6782	0.5632-0.6654	0.8301-0.9557		1.000-1.000		0.6347-0.7839		0.9505-0.9907	

VAL

									**LASSO**							
	Selected clinical Variables				Significant variables				Selected clinical variables				Significant variables			
	Complete case analysis	Imputation	Up-sampling	Imputation after up-sampling	Complete case analysis	Imputation	Up-sampling	Imputation after up-sampling	CCA	Imp	Upsamp and CCA	Imp after upsam	CCA	Imp	Upsamp and CCA	Imp after upsam
Accuracy	0.75	0.75	0.6444	0.7111	0.8333	0.8333	0.6923	0.6923	0.6975	0.5417	0.5417	0.5833	0.7708	0.7917	0.6875	0.7083
95% CI	0.6040-0.8636	0.604-0.8636	0.4878-0.7813	0.5569-0.8362	0.6978-0.9252	0.6978-0.9252	0.5243-0.8298	0.5243-0.9298	0.5375-0.8134	0.3917-0.6863	0.3917-0.6863	0.4321-0.7239	0.6269-0.8797	0.6501-0.8953	0.5375-0.8134	0.5594-0.8305
p-value	0.7045	0.7045	0.9869	0.8926	0.1976	0.1976	0.9049	0.9049	0.9347	0.9999	0.9999	0.9989	0.5799	0.4444	0.9347	0.8833
Balanced accuracy	0.5504	0.5504	0.6286	0.6714	0.6683	0.6364	0.6833	0.6833	0.6695	0.5749	0.5749	0.5381	0.6916	0.6413	0.7015	0.6511
Sensitivity	0.1818	0.1818	0.6	0.6000	0.3636	0.2727	0.6667	0.6667	0.7027	0.5135	0.5135	0.6216	0.8378	0.9189	0.6757	0.7568
Specificity	0.9189	0.9189	0.6571	0.7429	0.973	1	0.7000	0.7000	0.6364	0.6364	0.6364	0.4545	0.5455	0.3636	0.7273	0.5455
PPV	0.4000	0.4	0.3333	0.4000	0.8000	1	0.4000	0.4000	0.8667	0.8261	0.8261	0.7931	0.8611	0.8293	0.8929	0.8485
NPV	0.7907	0.7907	0.859	0.8667	0.8372	0.8222	0.8750	0.8750	0.3889	0.2800	0.2800	0.2632	0.5000	0.5714	0.4000	0.4000
AUC	0.6167	0.6388	0.6457	0.6571	0.7002	0.6192	0.7815	0.7926	0.6695	0.5749	0.5749	0.5318	0.6916	0.6413	0.7015	0.6511
AUC 95%CI	0.4311-0.8023	0.4552-0.8224	0.423-0.8491	0.4553-0.8589	0.526-0.8745	0.4151-0.8232	0.6345-0.9284	0.6435-0.9417	0.5028-0.8363	0.4050-0.7449	0.4050-0.7449	0.3646-0.7115	0.5260-0.8573	0.4857-0.7969	0.5437-0.8593	0.4816-0.8206

TEST

	Logistic Regression								LASSO								Random Forest							
	Selected clinical variables				Significant Variables				Selected clinical variables				Significant Variables				Selected clinical variables				Significant Variables			
	CCA	Imp	Up-sample	Imp with Up-sample	CCA	Imp	Up-sample	Imp with Up-sample	CCA	Imp	Up-sample	Imp with Up-sample	CCA	Imp	Up-sample	Imp with Up-sample	CCA	Imp	Up-sample	Imp with Up-sample	CCA	Imp	Up-sample	Imp with Up-sample
Accuracy	0.3800	0.6600	0.5600	0.6200	0.5800	0.6600	0.6400	0.6600	0.5800	0.2200	0.5400	0.5200	0.7000	0.7400	0.6800	0.7200	0.7200	0.7000	0.6400	0.7000	0.7000	0.7600	0.7000	0.7400
95% CI	0.2465-0.5283	0.5123-0.7879	0.4125-0.7001	0.4717-0.7535	0.4321-0.7181	0.5123-0.7879	0.4919-0.7708	0.5123-0.7879	0.4321-0.7187	0.1153-0.3596	0.3932-0.6819	0.3742-0.6634	0.5539-0.8214	0.5966-0.8537	0.5330-0.8048	0.5751-0.8377	0.5751 - 0.8377	0.5539 - 0.8214	0.4919 - 0.7708	0.5539 - 0.8214	0.5539 - 0.8214	0.6183 - 0.8694	0.5539 - 0.8214	0.5966-0.8537
p-value	1.0000	0.9616	0.9995	0.9912	0.9985	0.9616	0.9809	0.9616	0.9985	1.0000	0.9999	0.9999	0.8753	0.6977	0.9282	0.7987	0.79873	0.87529	0.9809	0.8753	0.8753	0.57668	0.8753	1
**Balanced accuracy**	**0.4781**	**0.5197**	**0.5395**	**0.6075**	**0.6952**	**0.5768**	**0.5921**	**0.5768**	**0.5526**	**0.4298**	**0.5548**	**0.5132**	**0.6031**	**0.5724**	**0.6469**	**0.6447**	**0.4737**	**0.4605**	**0.4781**	**0.5175**	**0.4890**	**0.5000**	**0.6031**	**0.6294**
Sensitivity (recall)	0.6667	0.2500	0.5000	0.5833	0.7500	0.4167	0.5000	0.4167	0.6053	0.0263	0.5263	0.5263	0.7895	0.8947	0.7105	0.7895	0.0000	0.0000	0.1667	0.1667	0.08333	0.0000	0.4167	0.4167
Specificity	0.2895	0.7895	0.5789	0.6316	0.5263	0.7368	0.6842	0.7368	0.5000	0.8333	0.5833	0.5000	0.4167	0.2500	0.5833	0.5000	0.9474	0.9211	0.7895	0.8684	0.89474	1.0000	0.7895	0.8421
PPV (precision)	0.2286	0.2727	0.2727	0.3333	0.3333	0.3333	0.3333	0.3333	0.7931	0.3333	0.8000	0.7692	0.8108	0.7907	0.8438	0.8333	0.0000	0.0000	0.2000	0.2857	0.2000	-	0.3846	0.4545
NPV	0.7333	0.7692	0.7857	0.8276	0.8696	0.8000	0.8125	0.8000	0.2857	0.2128	0.2800	0.2500	0.3846	0.4286	0.3889	0.4286	0.7500	0.7447	0.7500	0.7674	0.75556	0.7600	0.8108	0.8205
**AUC**	**0.5175**	**0.6360**	**0.6075**	**0.6184**	**0.6590**	**0.6524**	**0.6590**	**0.6546**	**0.5526**	**0.4298**	**0.5548**	**0.5132**	**0.6031**	**0.5724**	**0.6469**	**0.6447**	**0.4737**	**0.5746**	**0.4781**	**0.5033**	**0.4890**	**0.5000**	**0.6031**	**0.6557**
AUC 95%CI	0.3177-0.7174	0.4557-0.8162	0.4279-0.7871	0.4393-0.7976	0.4708-8471	0.4682-0.8366	0.4691-0.8489	0.4666-0.8427	0.3852-0.7200	0.3167-0.5429	0.3884-0.7212	0.3449-0.6814	0.4433-0.7629	0.4352-0.7095	0.4840-0.8099	0.4831-0.8064	0.4377 - 0.5097	0.368-0.781	0.3499-0.6063	0.3789-0.6276	0.3936 - 0.5845	0.500-0.500	0.4433-0.7629	0.4819-0.8295

Link S1. GitHub repository

https://github.com/kieranzu/TOASTIE_Predict/blob/main/toast_HN_241008.R
